# Associations between arterial oxygenation and oxidative stress markers in serum and bronchoalveolar lavage fluid of patients with severe brain injury

**DOI:** 10.3389/fneur.2026.1794885

**Published:** 2026-07-21

**Authors:** Dorota Siwicka, Sylwia Terpilowska, Agnieszka Kubik-Komar, Wojciech Dabrowski

**Affiliations:** 1 Department of Anaesthesiology and Intensive Care Medical University of Lublin, Lublin, Poland; 2 Department of Surgical Medicine with the Laboratory of Medical Genetics, Collegium Medicum, Jan Kochanowski University, Lublin, Poland; 3 Department of Applied Mathematics and Computer Science, University of Life Sciences in Lublin, Lublin, Poland

**Keywords:** bronchoalveolar lavage fluid, mechanical ventilation, oxidative stress markers, oxygen therapy, severe brain injury

## Abstract

**Background:**

Severe isolated brain injury can trigger systemic oxidative and inflammatory responses extending to distant organs, including the lungs. Supplemental oxygen therapy is routinely used in neurocritical care, but elevated arterial oxygenation may be associated with redox alterations.

**Objectives:**

The primary objective was to characterize temporal dynamics of systemic and pulmonary oxidative stress in patients with severe traumatic brain injury during the first 7 days. Secondary objectives were to assess associations between oxidative stress marker concentrations and arterial oxygenation parameters, clinical severity scores, and 28-day mortality. This was an exploratory study and no causal inferences are drawn.

**Design:**

Prospective observational cohort study.

**Setting:**

First-level trauma centre intensive care unit over 12 months.

**Patients:**

Forty mechanically ventilated adult patients with isolated severe brain injury were enrolled.

**Interventions:**

No therapeutic intervention was applied. Standard intensive care management was maintained according to institutional protocols.

**Main outcome measures:**

Primary outcomes were concentrations of superoxide dismutases (SOD), catalase (CAT), glutathione peroxidase (GPx), malondialdehyde (MDA), and 8-hydroxy-2′-deoxyguanosine (8-OHdG) measured in serum and bronchoalveolar lavage fluid at three time points during the first 7 days. Secondary outcomes included correlations with arterial oxygen tension (PaO_2_), inspired oxygen fraction (FiO_2_), clinical severity scores, and 28-day mortality.

**Results:**

Antioxidant enzyme concentrations decreased and oxidative damage marker concentrations increased progressively (*p* < 0.001 for all). Repeated-measures correlation analysis showed that lower PaO_2_ values were associated with higher 8-OHdG levels in serum (*r* = −0.64) and bronchoalveolar lavage fluid (*r* = −0.55), and with lower SOD and CAT concentrations. The association between PaO_2_ and MDA was more complex: in mixed-effects models, PaO_2_ was positively associated with antioxidant enzyme concentrations and serum MDA concentration, suggesting that the relationship between oxygenation and lipid peroxidation is not straightforward. In an exploratory threshold-based analysis, PaO₂ values exceeding 100 mmHg at the earliest time point were associated with higher 28-day mortality (*p* < 0.05); however, this finding was not supported by a fully adjusted prognostic model and should be interpreted cautiously.

**Conclusion:**

Severe isolated brain injury is associated with progressive systemic and pulmonary oxidative stress. The relationship between arterial oxygenation and oxidative stress appears complex and may depend on timing and oxygen exposure levels.

## Highlights

- Severe isolated brain injury is associated with systemic and pulmonary oxidative stress.
- Oxidative damage increased over time, whereas antioxidant marker concentrations decreased.
- The relationship between PaO₂ and oxidative stress was complex and differed according to the analytical approach and time point.
- Early PaO₂ values above the predefined exploratory threshold of 100 mmHg were associated with increased 28-day mortality in an unadjusted exploratory analysis.
- These findings generate hypotheses regarding oxygenation-associated redox alterations in neurocritical care and require confirmation in larger, fully adjusted studies.

## Introduction

Severe brain injury is one of the most prevalent causes of chronic physical disabilities worldwide. Brain damage results from primary insults; the subsequent biochemical cascades are collectively termed secondary brain injury. Recent analyses have shown that oxidative stress and mitochondrial dysfunction are important mediators of secondary injury.

Additionally, critically ill patients with isolated severe brain injury frequently receive mechanical ventilation with supplemental oxygen therapy, thus supplying oxygen to damaged cells and decreasing the level of secondary injury (1). However, oxygen therapy should be titrated carefully because both hypoxemia and hyperoxemia are associated with worse outcomes in some clinical studies of patients with traumatic brain injury, aneurysmal subarachnoid hemorrhage, or after cardiac arrest (2–4).

Oxidative stress is the instability between prooxidant mechanisms and antioxidant defenses. It is caused by an imbalance between the production and accumulation of reactive oxygen species (ROS) in cells and tissues and the ability of the mechanisms to remove and detoxify them. ROS are generally involved in redox signaling; however, in specific situations, they may lead to oxidative injury. It is often both the cause and the result of injury.

Oxygen (O_2_) toxicity is associated with uncontrolled production of reactive oxygen species (ROS) and disturbance of the balance between free radicals and antioxidants (5). In a review article, Chen et al. documented the important role of targeting mechanisms of ferroptosis and oxidative stress in protection strategies of blood–brain barrier disruption (6). Blood factors such as thrombin, C-reactive protein (CRP), and fibrinogen may initiate the immune system and oxidative stress in the central nervous system (CNS), triggering the breakdown of the blood–brain barrier (BBB) integrity (7). In addition, vascular disruption during stroke leads to oxidative stress and impaired nutrient supply, ultimately activating metalloproteinases (MMPs) and disrupting the blood–brain barrier (BBB) (8–10). Traumatic brain injury (TBI) also draws attention to the infiltration of the brain parenchyma by blood factors and circulating immune cells (11).

The concentration of ROS in cells should be kept relatively low to maintain homeostasis. Therefore, cells have developed various mechanisms to alter their integrity against the action of free radicals. Increased concentrations of ROS have detrimental effects on key cellular structures, such as proteins, lipids, and nucleic acids. Reactive oxygen species destroy the cell membrane structure, increasing its fluidity and permeability. Importantly, elevated levels of oxidative stress decrease antioxidant defenses, stimulate autophagy/mitophagy processes, affect cell death, and stimulate inflammation in the lungs.

A growing body of evidence indicates that elevated ROS generation and impaired mitochondrial function are characteristic of severe ARDS and contribute to the exacerbation of inflammation (12). During lung injury, critical tissue damage and intensification of inflammatory processes impact the endogenous antioxidant system, which becomes overwhelmed. Different types of cells, including alveolar epithelial cells, endothelial cells, neutrophils, macrophages, and eosinophils, overproduce ROS, resulting in cell death (13).

We hypothesized that isolated severe brain injury is associated with systemic and pulmonary oxidative stress and that oxygenation parameters may be associated with redox alterations. Given the exploratory and observational design of this study, the analyses were intended to generate hypotheses regarding the temporal dynamics of oxidative stress and its associations with oxygenation parameters, rather than to establish causal relationship.

The primary objective of this exploratory study was to characterize the temporal concentrations of systemic and pulmonary oxidative stress and antioxidant markers in patients with isolated severe traumatic brain injury during the first 7 days after injury.

Secondary objectives were to assess associations between oxidative stress markers and arterial oxygenation parameters (PaO₂, FiO₂), clinical severity indices, and 28-day mortality.

## Materials and methods

### Study overview

This prospective observational cohort study was conducted at the First Level Trauma Centre in Clinical Hospital No. 4 in Lublin, Poland.

The study procedures were conducted in accordance with the ethical principles of the Declaration of Helsinki and the applicable regulatory requirements and were approved by the Bioethics Committee of the Medical University in Lublin, Poland (Chairperson: Prof. Andrzej Głowniak, approval date: 02.10.2017, reference no. KE-0254/210/2017). Written informed consent was obtained from the patients’ legal representatives prior to study inclusion.

### Participants

The inclusion criteria were as follows: isolated brain injury, presentation to the Intensive Care Unit (ICU), receiving mechanical ventilation support during the initial 24 h, and a computed tomography CT scan <24 h after injury. Exclusion criteria were pregnancy, age under 18 years, neoplastic, pulmonary, or hepatorenal chronic disease history, drug intoxication and prior transplant recipients, lung contusions, chest injury, acute heart failure with pulmonary edema, and abdominal injury. The final study population included 40 patients.

### Clinical variables

All patients were recruited to the study between 2018 and 2021 and diagnoses of brain injury were defined according to the International Classification of Diseases and Related Health Problems, 10th Revision (14). Patients were assessed by interviews and medical record review at the emergency department and ICU admission. Demographic data, medical history, and clinical and injury history were collected.

Brain injury was classified according to neuroradiological findings on admission computed tomography (CT). The Rotterdam CT score was selected to classify radiographic severity during admission to the ICU.

Cranial surgery and intracranial pressure monitoring were also evaluated. In selected patients with diagnosed intracranial space-occupying lesions, that is, subdural and/or epidural hematomas, craniotomy or craniectomy was performed at the discretion of the neurosurgeon.

TBI severity was classified using the Glasgow Coma Scale (GCS), Rotterdam CT score, and Marshall CT classification.

### Monitoring and treatment

Patients with severe brain injury between July 2018 and January 2021 involved in the study were sedated and managed according to the latest guidelines (15). Monitoring and treatment techniques have been previously described (16). All patients with severe isolated brain damage were mechanically ventilated according to strategies described recently (17, 18). The PEEP levels were set between 5 and 8 cm H_2_O to optimise oxygenation.

### Sample collection

Serum and BALF from the subjects were collected at three time points: within 6–8 h after isolated brain injury (A) and on days 3 (B) and 7 (C).

The bronchoscopy and BALF procedures were previously described in our protocols (19, 20). The collected two millilitres of BALF were centrifuged to separate the fluid from the cells (1,900 rpm/10 min/room temperature). The collected 1–2 mL of whole blood samples, were allowed to clot at room temperature, usually for 30–60 min. Samples were centrifuged (4,000 RPM for 15 min) to separate serum from the cells. The serum was stored at − 80 °C for further study.

### Biochemical analyses

The concentrations of oxidative damage markers, malondialdehyde (MDA) and 8-hydroxy-2′-deoxyguanosine (8-OHdG), and antioxidant defense markers, superoxide dismutases (SOD), catalase (CAT), and glutathione peroxidase (GPx), were determined in serum and bronchoalveolar lavage fluid (BALF) using commercially available enzyme-linked immunosorbent assay (ELISA) kits (Cloud-Clone Corp., USA), according to the manufacturer’s instructions. The available manufacturer documentation does not specify that the assay is selective for SOD1 or provide isoform-specific cross-reactivity data for SOD3. Therefore, the measured marker is reported as SOD protein concentration rather than as SOD1 concentration.

The data from the test manufacturer, detection range, and sensitivity of the assays are presented in Supplementary Table 1. Serum and BALF supernatant were used for all analyses.

Prior to analysis, serum and BALF supernatant samples were diluted 50-fold using the sample diluent provided with the ELISA kits, according to the manufacturer’s instructions, and 100 μL of the diluted material was added to each well. Each sample was measured in triplicate according to the manufacturer’s protocol, and the mean value of the replicate measurements was used for statistical analysis. Calibration curves were prepared using manufacturer-provided standards specific for each analytic included in the ELISA kits. When more than one ELISA plate was required, calibration curves were prepared separately for each plate using the manufacturer-provided standards. The stock standard solution had a concentration of 80 ng/mL and was serially diluted to obtain concentrations of 10, 5, 2.5, 1.25, 0.624, 0.312, and 0.156 ng/mL. Calibration curve diagrams together with corresponding R^2^ values were included in Supplementary Figures 1, 2. The intra-assay coefficient of variation was <10%, while the inter-assay coefficient of variation was within the range specified by the manufacturer’s data.

Absorbance was measured using a microplate reader (Thermo Scientific Multiscan). The assays quantify protein concentrations rather than enzymatic activity; therefore, all results are reported as concentrations.

### Study variables

For the purposes of this exploratory analysis, PaO₂ values were classified as below the reference range (PaO₂ < 75 mmHg), within the predefined reference range (PaO₂ 75–100 mmHg), and above the predefined exploratory threshold (PaO₂ > 100 mmHg). Because this threshold is lower than that used in many previous studies of hyperoxemia, the term hyperoxemia is used cautiously and operationally in this manuscript (20).

Selected blood parameters, such as inflammatory process markers, morphology, and blood gas analyses, were performed as described previously (20). Oxidative stress markers were categorized into oxidative damage markers (MDA, 8-OHdG) and antioxidant defense enzymes (SOD, CAT, GPx), allowing a more structured interpretation of redox imbalance.

Additionally, extravascular lung water (EVLWI) and pulmonary vascular permeability index (PVPI) were documented.

The chosen study variables were correlated between oxidative stress marker concentrations in serum and BALF, and selected score systems such as the Glasgow Coma Score (GCS), Rotterdam computed tomography score (CTS) care system, and Marshall score.

Due to the small number of patients in the Marshall grade II and III groups (II, *n* = 5; III, *n* = 3), these categories were combined for the purposes of statistical analysis. This decision was based on the relatively minor clinical differences between the two groups in terms of post-traumatic changes as well as their similar prognostic significance. This approach allowed the maintenance of sufficient statistical power in the comparative analysis of biomarker levels across Marshall scale categories. Notably, this modification did not affect the final conclusions of the analysis. Mortality was evaluated on Day 28.

### Statistical analysis

This was an exploratory study without a formal sample size calculation. Forty consecutive patients who met the eligibility criteria were included in the study.

Considering both factors of repeated measurements, as well as the fact that marker values were not normally distributed for most repetitions, the study proposed the use of the nonparametric marginal model for the LD-F2 design with material (BALF/serum) and repetition (A, B, C) as sub-plot factors for this comparison (21). The relative treatment effect (RTE) and ANOVA-type statistic were calculated for each of the two sub-plot factors as well as their interactions.

For *post hoc* comparisons between time points, nonparametric relative treatment effects were estimated using the multiple contrast procedure implemented in the R package nparcomp, applying Tukey-type contrasts with simultaneous confidence intervals (22).

To assess associations between physiological parameters and oxidative stress markers while accounting for the longitudinal structure of the data, linear mixed-effects models were applied (23). For each oxidative stress marker (SOD, CAT, GPx, MDA, 8-OHdG) and biological compartment (serum, BALF), separate models were constructed including arterial oxygenation parameters (PaO₂, FiO₂), extravascular lung water index (ELWI), and pulmonary vascular permeability index (PVPI) as fixed effects. Time (A, B, C) was included as a categorical fixed effect, and patient ID was included as a random intercept to account for within-subject correlations (24).

Due to skewed distributions, marker concentrations were log-transformed prior to modeling.

For comparison and descriptive assessment of within-subject linear associations across time points, repeated-measures correlation analysis was additionally performed (25).

The Welch’s *t*-test was used to assess differences in the values of individual markers between patients who survived and those for whom oxidative stress resulted in death. For this purpose, the average values of the markers from three measurements (A, B, and C) were determined, and these data were subjected to statistical analysis.

The average marker values (means of A, B, and C) were used to compare the variation in marker values among patients marked on individual scales. For this purpose, a one-way ANOVA was applied, and in the case of statistically significant differences, a Tukey *post hoc* test was performed.

The calculations and plots were performed using R and Statistica v13 software (26).

## Results

### Characteristics of the patients

Baseline characteristics of the study population are presented in Supplementary Table 2. The cohort comprised 40 patients (24 male, 16 female) with a mean age of 45 ± 15 years. The median GCS score on ICU admission was 4.5. According to the Marshall CT classification, the majority of patients were classified as grade V (*n* = 19), followed by grade IV (*n* = 8) and grade VI (*n* = 5); all five patients in grade VI were non-survivors. The 28-day mortality rate was 40% (16/40). Full demographic and clinical data for this cohort have been reported previously (20).

### Oxidative stress factors concentration and temporal changes

The concentrations of selected antioxidant response factors (SOD, CAT, GPx) and oxidative damage markers (MDA, 8OHdG) in the serum and BALF at three time points were evaluated.

**Figure 1:**
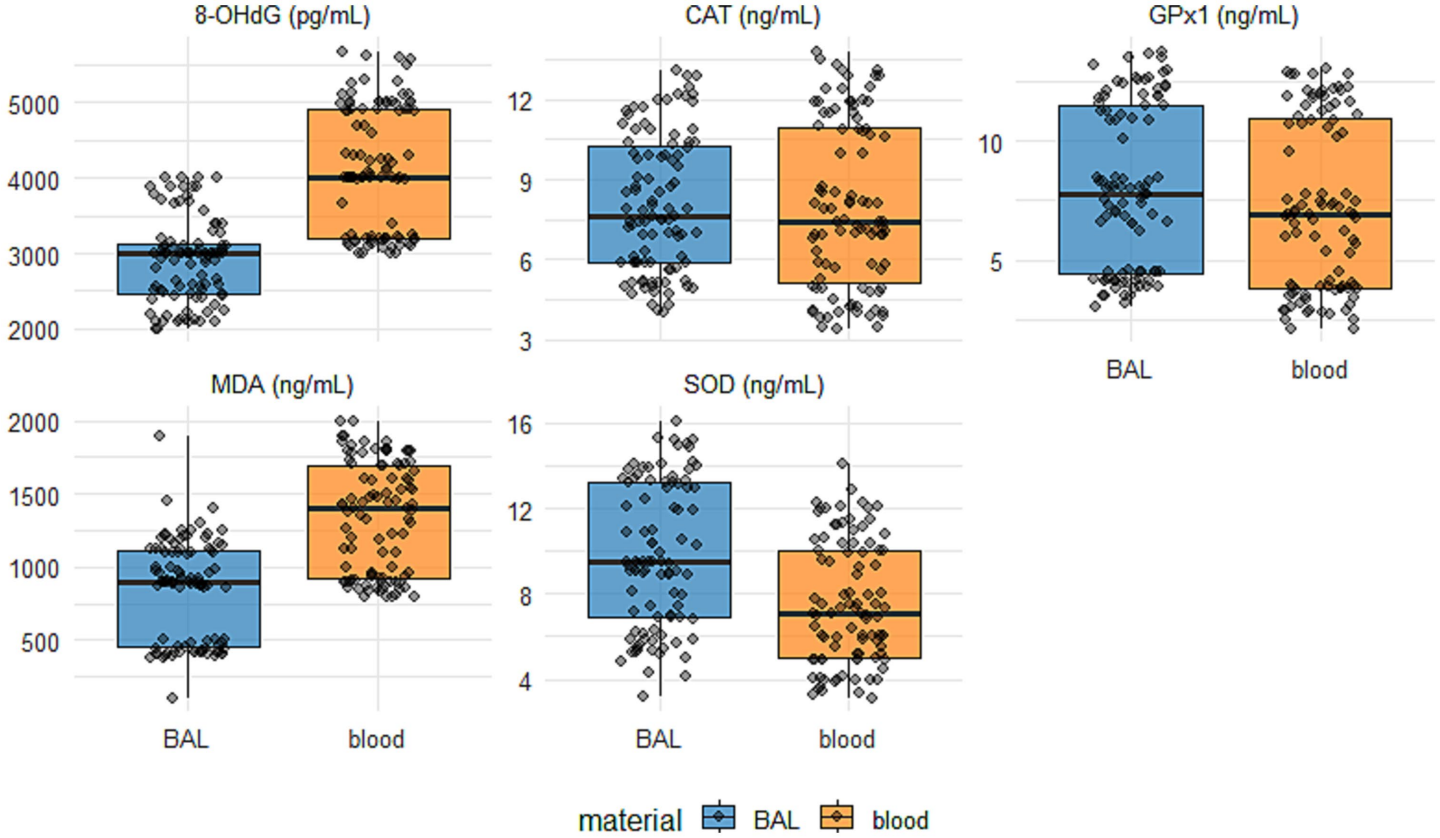
The concentrations of oxidative stress markers hydroxydeoxyguanosine (8-OHdG,) malondialdehyde (MDA) and antioxidants enzymes: superoxide dismutases (SOD), catalase (CAT), and glutathione peroxidase 1 (GPx1), in the BALF and serum of patients with severe brain injury. The parallel decline in SOD, CAT, and GPx over time, accompanied by a progressive rise in MDA and 8-OHdG.

The ANOVA-type test showed no significant difference between the serum and BALF concentrations of antioxidant marker CAT (*p* = 0.085). There were significant differences between the concentrations of antioxidant enzymatic markers SOD and GPx [higher levels in BALF than in serum (*p* < 0.001)] and for oxidative damage markers 8-OHdG and MDA (higher levels in serum, *p* < 0.001). The Tukey-type contrasts for changes of markers showed a statistically significant decrease in selected antioxidant factors such as SOD, CAT, and GPx (*p* < 0.001) between three-time points A, B, and C. Importantly, a statistically significant increase in 8-OHdG and MDA (*p* < 0.001) were documented between mentioned time points. The parallel decline in SOD, CAT, and GPx over time, accompanied by a progressive rise in MDA and 8-OHdG, suggests depletion of endogenous antioxidant reserves rather than recovery, indicating persistent oxidative pressure throughout the first week after injury. The concentration of selected antioxidants and oxidative stress markers are presented in Figure 1.

### Associations between physiological parameters and oxidative stress markers

#### Association with oxygenation

The results of the mixed-effects model analysis examining associations between physiological parameters and oxidative stress markers are presented in Supplementary Table 3.

In serum, ELWI was positively associated with concentration of oxidative damage marker 8-OHdG and negatively associated with concentration of antioxidant marker GPx. No significant associations were observed between ELWI and other serum markers. In BALF, ELWI was positively associated with MDA concentrations, whereas no other significant associations were observed. PVPI was not significantly associated with oxidative stress markers in either biological compartment.

In serum, PaO₂ was positively associated with antioxidant enzyme concentrations (CAT, GPx and SOD) as well as with MDA concentration. In BALF, PaO₂ was positively associated with CAT and GPx.

FiO₂ showed a significant positive association with SOD concentration in serum, while other associations did not reach statistical significance.

**Figure 2:**
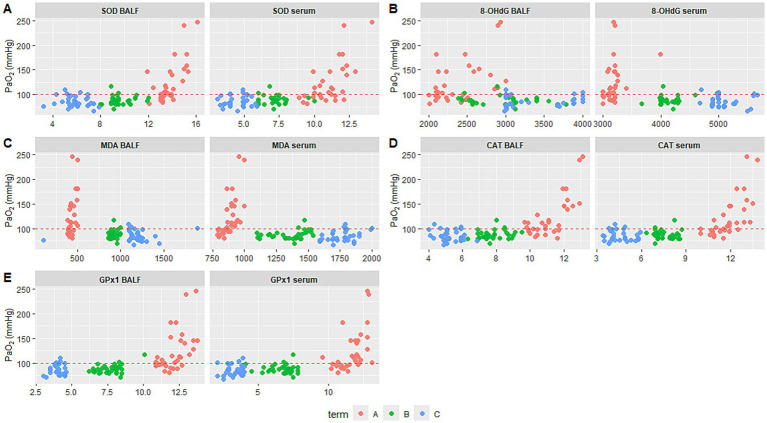
Repeated-measures correlations between arterial oxygen tension (PaO_2_) and oxidative stress markers and antioxidant enzyme concentrations in serum and bronchoalveolar lavage fluid (BALF). Correlations are shown for **(A)** superoxide dismutase (SOD), **(B)** 8-hydroxy-2′-deoxyguanosine (8-OHdG), **(C)** malondialdehyde (MDA), **(D)** catalase (CAT), and **(E)** glutathione peroxidase 1 (GPx1). Measurements were obtained at three time points: within 6–8 h after injury (red), on day 3 (green), and on day 7 (blue). Each point represents an individual patient measurement. The red dashed horizontal line indicates a PaO_2_ value of 100 mmHg.

Repeated-measures correlation analysis demonstrated strong within-subject associations between oxygenation parameters and oxidative stress markers, and are presented in Table 1, Figure 2.

**Table 1:** Repeated measures correlations between selected oxidative stress markers and clinical variables across the three time points.

Selected markers	Material	FiO_2_	PaO_2_	ELWI	PVPI
SOD	BALF	0.12	0.666***	−0.747**	−0.57**
SOD	serum	0.24	0.692***	−0.719**	−0.522**
8-OHdG	BALF	−0.201	−0.554***	0.689**	0.483**
8-OHdG	serum	−0.202	−0.644***	0.781**	0.509**
CAT	BALF	0.227	0.717***	−0.754**	−0.531**
CAT	serum	0.194	0.697***	−0.749**	−0.51**
GPx	BALF	0.221	0.668**	−0.766**	−0.512**
GPx	serum	0.227	0.69**	−0.788**	−0.539**

SOD, CAT, and GPx concentrations in both BALF and serum correlated positively with PaO_2_ (*r* = 0.666–0.717, *p* < 0.001) and negatively with ELWI (*r* = −0.719 to −0.788, *p* < 0.01) and PVPI (*r* = −0.510 to −0.539, *p* < 0.01), whereas no significant association with FiO_2_ was observed for these markers. In contrast, 8-OHdG levels in both compartments showed significant negative correlations with PaO_2_ (BALF: *r* = −0.554, *p* < 0.001; serum: *r* = −0.644, *p* < 0.001) and positive correlations with ELWI (*r* = 0.689–0.781, *p* < 0.01) and PVPI (*r* = 0.483–0.509, *p* < 0.01), with no significant correlation detected for FiO_2_. These results indicate a significant association between antioxidant enzyme activity and clinical parameters of lung function. Significance * < 0.05, ** < 0.01, *** < 0.001.

The PaO₂ value showed a significant negative correlation with 8-OHdG levels in both the serum (*r* = −0.64) and BALF (*r* = −0.55), indicating that DNA oxidative damage increased as oxygenation decreased. Similarly, ELWI was negatively correlated with antioxidant enzyme activities and positively correlated with 8-OHdG.

Conversely, positive correlations were observed between PaO₂ and antioxidant enzyme activities, including SOD (*r* = 0.67), CAT (*r* = 0.72), and GPx, particularly in the BALF samples. The direction of these associations was largely consistent with the mixed-effects model results, particularly for PaO₂ and ELWI. However, although PVPI demonstrated significant correlations in the repeated-measures analysis, it did not remain an independent predictor in the mixed-effects models.

In contrast, 8-OHdG levels in both compartments showed significant negative correlations with PaO_2_ (BALF: *r* = −0.554, *p* < 0.001; serum: *r* = −0.644, *p* < 0.001) and positive correlations with ELWI (*r* = 0.689–0.781, *p* < 0.01) and PVPI (*r* = 0.483–0.509, *p* < 0.01), with no significant correlation detected for FiO_2_. These results indicate a significant association between antioxidant enzyme activity and clinical parameters of lung function.

There were no statistically significant relationships between FiO_2_ and the concentration of selected markers. Importantly, there were significant positive correlations between PaO_2_/FiO_2_ during ICU admission and the values of all markers at time point A, except for 8-OHdG BALF. The strongest correlation between the studied features and markers was observed for CAT in the serum.

**Figure 3:**
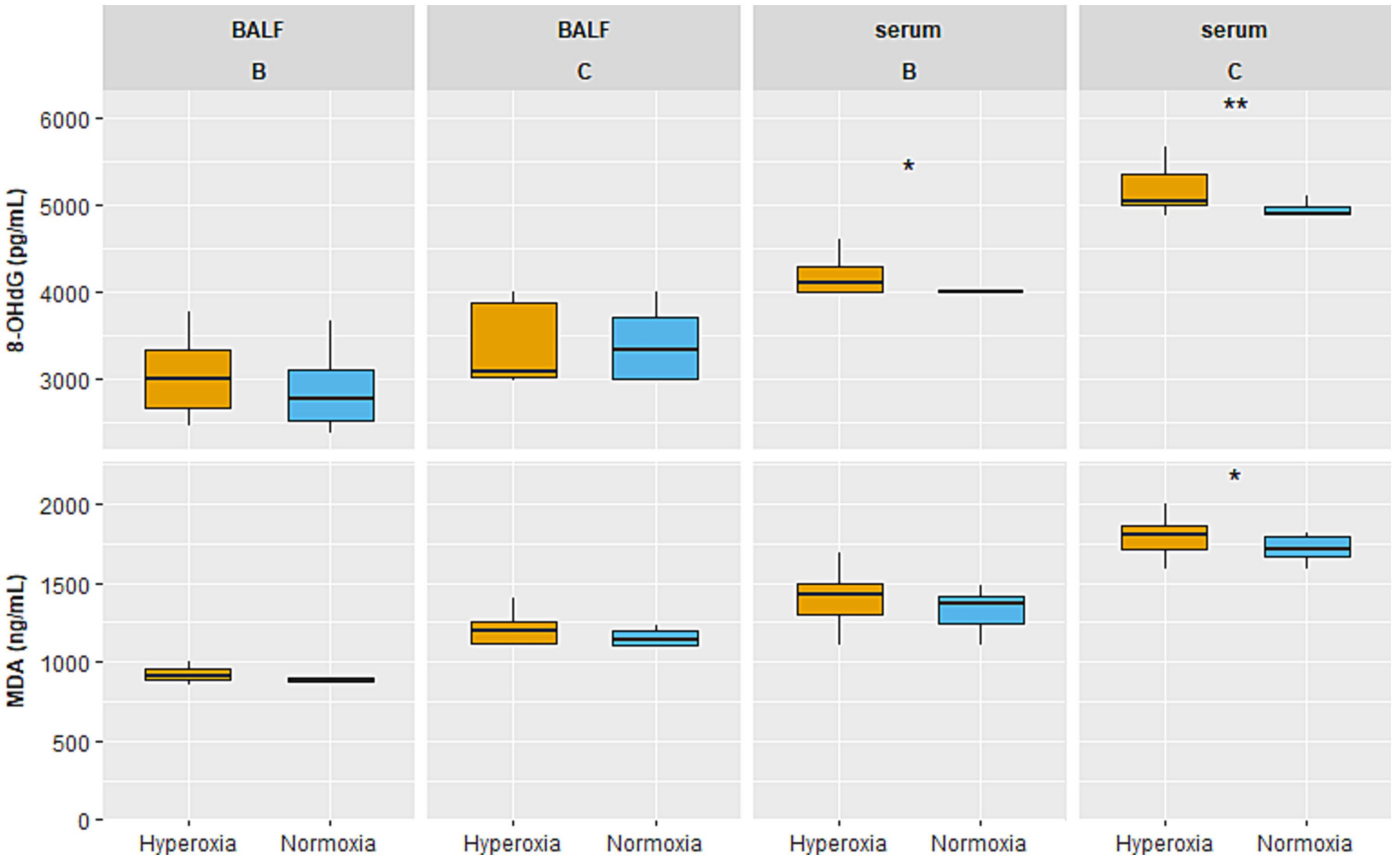
Concentrations of oxidative damage markers hydroxydeoxyguanosine (8-OHdG) malondialdehyde (MDA) in bronchoalveolar lavage fluid (BALF) and serum in patients with PaO₂ above and below the predefined exploratory threshold of 100 mmHg (PaO₂ > 100 mmHg vs. PaO₂ ≤ 100 mmHg) at time points B and C (days 3 and 7).

The concentrations of selected oxidative damage markers (8-OHdG and MDA) in bronchoalveolar lavage fluid (BALF) and serum among patients with PaO₂ above and below the predefined exploratory threshold of 100 mmHg are presented in Figure 3. Furthermore, Table 2 presents the results of the Mann–Whitney *U* test comparing oxidative stress marker concentrations between patients with and without hyperoxia, while Table 3 summarizes the Spearman correlations between the arterial oxygen partial pressure to inspired oxygen fraction ratio (PaO_2_/FiO_2_) at ICU admission and selected oxidative stress markers.

**Table 2:** Mann–Whitney *U* test results comparing oxidative stress biomarkers concentration between patients with and without hyperoxia (PaO₂ ≥ 100 mmHg vs. PaO₂ < 100 mmHg) at three time points (A, B, C) in bronchoalveolar lavage fluid (BALF) and serum.

Material	Time points		BAL	SERUM
Damage markers	8-OHdG	A	**0.03663**	0.09006
		B	0.17397	**0.04898**
		C	0.41542	**0.00418**
	MDA	A	**0.01837**	**0.00005**
		B	0.07593	0.11940
		C	0.07268	**0.02908**
Antioxidant responses markers	CAT	A	**0.03532**	**0.00041**
		B	0.66798	0.82960
		C	0.51746	0.21472
	GPx	A	**0.00565**	**0.00304**
		B	0.90675	0.17221
		C	**0.03129**	**0.00103**
	SOD	A	**0.02550**	**0.02550**
		B	0.54000	0.88600
		C	0.61150	0.55480

Values represent *p*-values. Statistically significant results (*p* < 0.05) are marked in bold.

**Table 3:** A Spearman correlation between the ratio of partial pressure of oxygen in arterial blood (PaO_2_) to the fraction of inspiratory oxygen concentration (FiO_2_) during admission to ICU and selected markers.

	SOD	SOD	8-OHdG BAL	8-OHdG	MDA BAL	MDA	CAT	CAT	GPx	GPx
	BAL	SERUM		SERUM		SERUM	BAL	SERUM	BAL	SERUM
PaO_2_/FiO_2_ ratio	0.493*	0.415*	0.224	0.368*	0.438*	0.660*	0.475*	0.691*	0.544*	0.449*

*p < 0.05.

### Relationship of selected factors in serum and in BALF and score systems

As previously reported non-survivors had a significantly lower GCS score and a higher Rotterdam CT score (20).

### Rotterdam CT scale

There were significant differences in the average marker values between the patients placed on the individual levels of the Rotterdam scale for serum SOD (*F* = 4.95, *p* = 0.007) and 8-OHdG (*F* = 3.27, *p* = 0.03).

**Figure 4:**
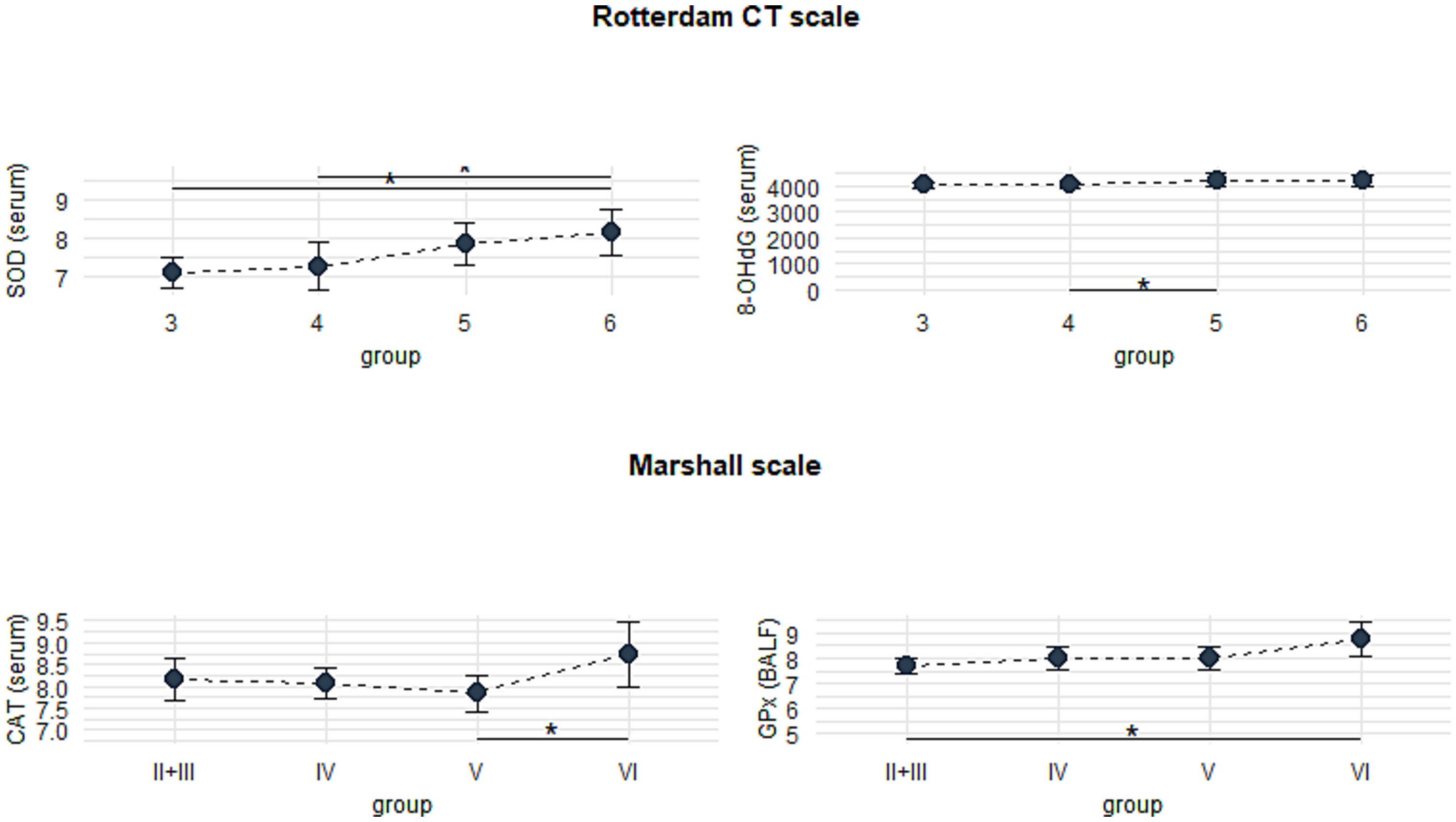
Comparison of oxidative stress markers across patient groups classified according to the Rotterdam CT and Marshall CT scales. Serum superoxide dismutases (SOD) was significantly lower in Rotterdam grade 6 than in grades 3 and 4 (*F* = 4.95, *p* = 0.007; post-hoc *p* = 0.022 and *p* = 0.029, respectively), while serum hydroxydeoxyguanosine (8-OHdG) was higher in grade 5 than grade 4 (*F* = 3.27, *p* = 0.030; post-hoc *p* = 0.049). Within the Marshall scale, serum catalase (CAT) was lower in grade VI versus grade V (*F* = 3.58, *p* = 0.020; post-hoc *p* = 0.025), and BALF glutathione peroxidase (GPx) was lower in grade VI versus grades II + III (*F* = 3.25, *p* = 0.026; post-hoc *p* = 0.014).

The post-hoc test results indicated significant differences in blood SOD levels between patients in groups 3 and 4 and those in group 6 (*p* = 0.022 and *p* = 0.029, respectively), as well as significant differences in blood 8-OHdG levels between patients in groups 4 and 5 (*p* = 0.049) Comparison of oxidative stress markers across patient groups classified according to the Rotterdam CT and Marshall CT scales is presented in Figure 4.

### Marshall scale

There were statistically significant differences in the average values of serum CAT (*F* = 3.58, *p* = 0.02) and BALF GPx (*F* = 3.25, *p* = 0.026) between patients placed at different levels of the Marshall scale. The post-hoc test revealed significant differences in serum CAT (*F* = 3.58, *p* = 0.026) between patients from groups V and VI (*p* = 0.025). There were significant differences in the average GPx values (BALF; *F* = 3.99, *p* = 0.017) between patients in groups II+III and VI (*p* = 0.014; Figure 4).

### Relationship of selected factors in serum and BALF according to mortality

**Figure 5:**
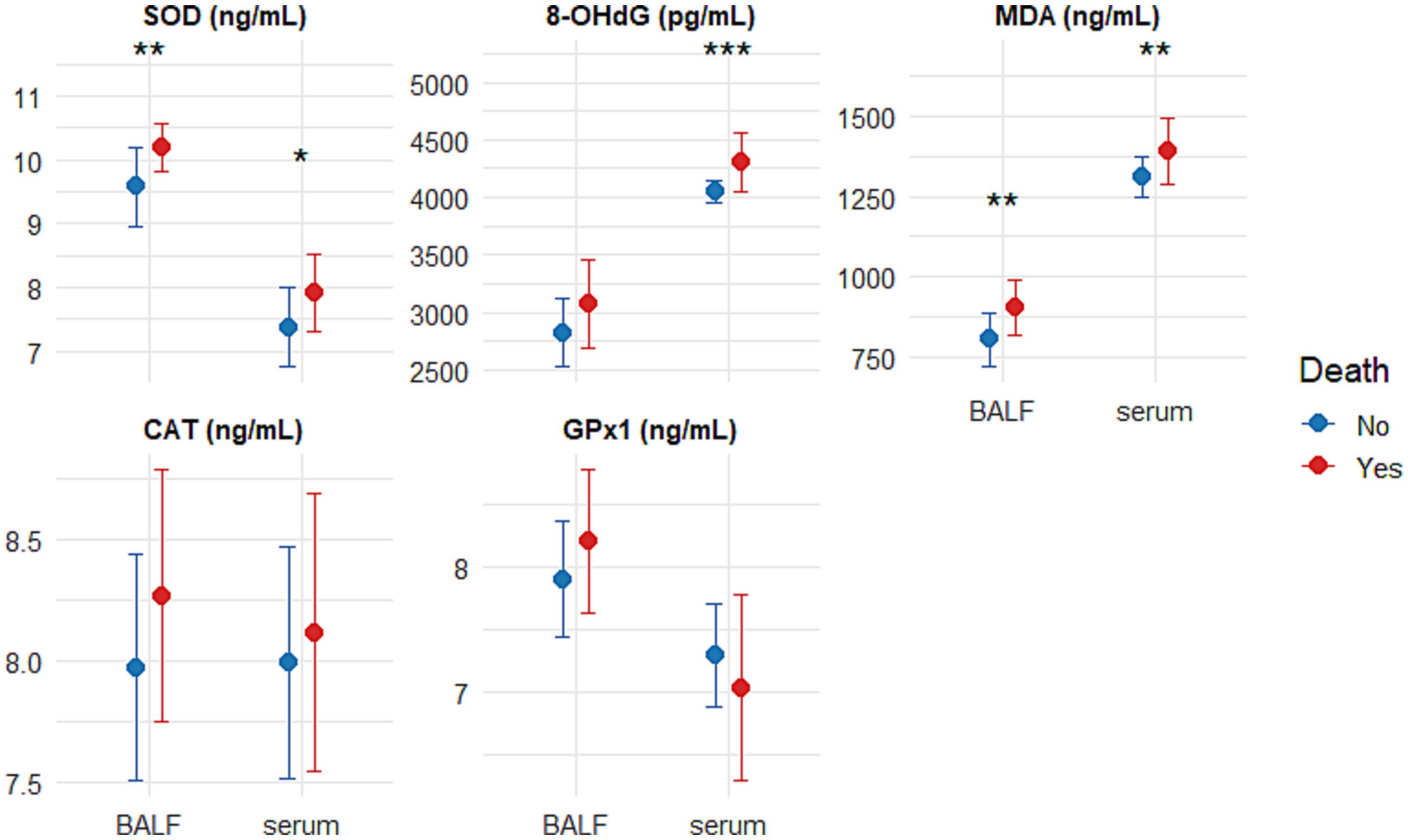
Serum and BALF (bronchoalveolar lavage fluid) concentrations of selected oxidative stress markers in surviving and non-surviving patients. Non-survivors exhibited significantly higher mean superoxide dismutases (SOD) levels in both BALF (*p* = 0.007) and serum (*p* = 0.032), higher malondialdehyde MDA levels in both compartments (BALF: *p* = 0.004; serum: *p* = 0.009), and higher serum hydroxydeoxyguanosine (8-OHdG,) concentrations (*p* < 0.001). No significant differences were observed for catalase (CAT) or glutathione peroxidase (GPx) in either compartment. The difference in BALF 8-OHdG between groups did not reach statistical significance (*p* = 0.056).

The Welch’s *t*-test was applied to compare mean marker concentrations (averaged across time points A, B, and C) between survivors and non-survivors. Non-survivors had significantly higher mean SOD concentrations in both BALF (*p* = 0.007) and serum (*p* = 0.032), significantly higher MDA concentrations in both BALF (*p* = 0.004) and serum (*p* = 0.009), and significantly higher serum 8-OHdG concentrations (*p* < 0.001). No statistically significant differences were observed for CAT or GPx in either compartment. The difference in BALF 8-OHdG between survivors and non-survivors did not reach statistical significance (*p* = 0.056), although the *p*-value was marginally above the threshold; this finding warrants further investigation in larger cohorts. Serum and BALF concentrations of selected oxidative stress markers in surviving and non-surviving patients is presented in Figure 5.

**Figure 6:**
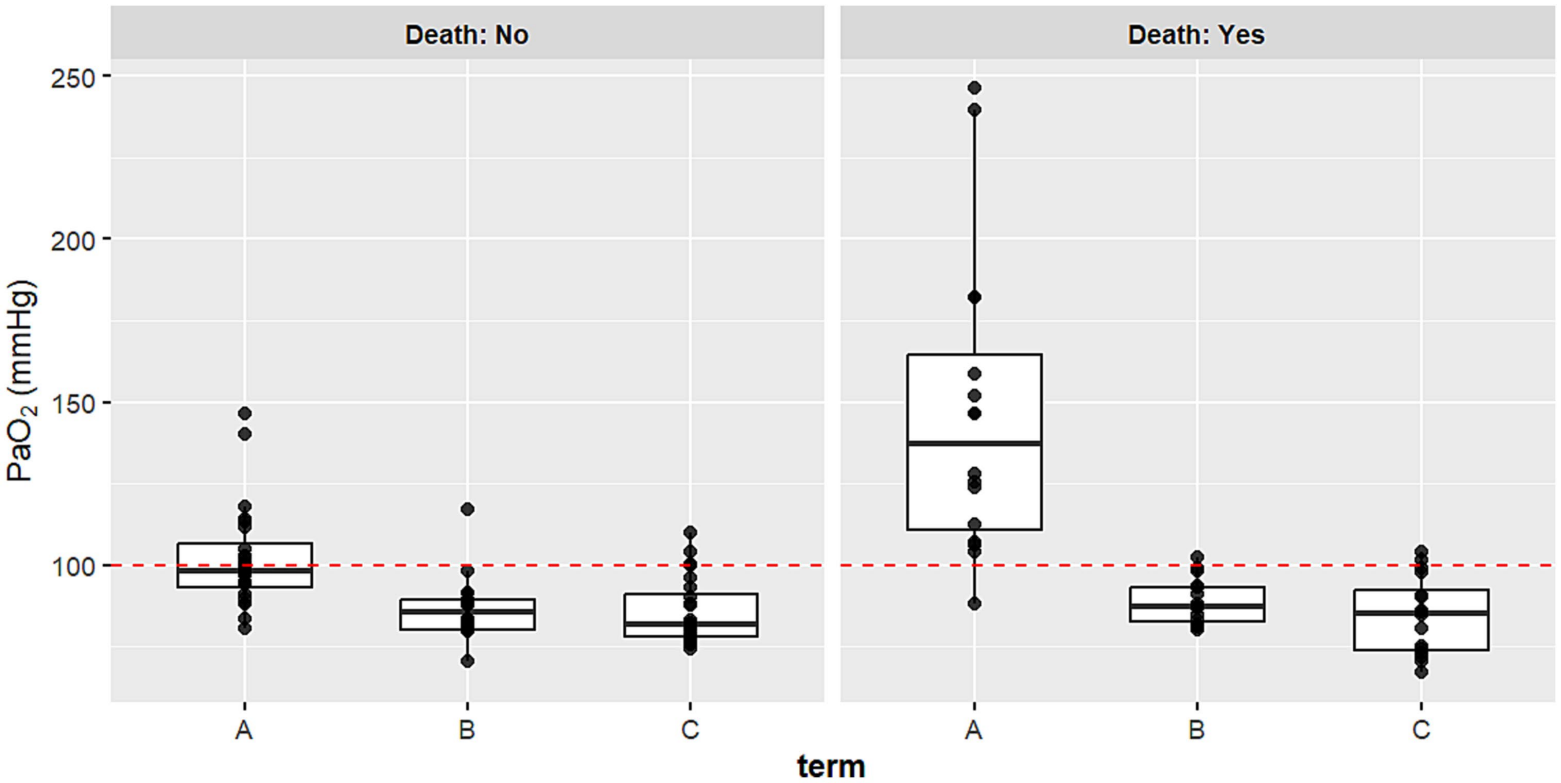
Correlation between PaO₂ (partial pressure of oxygen) and 28-day mortality at time points A, B, and C. Repeated-measures correlation analysis demonstrated that lower PaO₂ values were associated with higher hydroxydeoxyguanosine (8-OHdG) levels and lower antioxidant marker concentrations. Threshold-based analysis revealed that PaO₂ values exceeding 100 mmHg at the earliest time point A were associated with increased 28-day mortality (*p* < 0.05).

Repeated-measures correlation analysis showed that lower PaO₂ values were associated with higher 8-OHdG levels and lower antioxidant marker levels. However, in threshold-based analyses, PaO₂ values above 100 mmHg at the earliest time point A were associated with increased 28-day mortality, (*p* < 0.05). Correlation between PaO₂ and 28-day mortality at time points A, B, and C is presented in Figure 6. As no multivariable adjustment was performed, these findings should be considered exploratory.

## Discussion

The primary finding of the study was a significant decrease in the antioxidant enzymatic components, such as SOD, CAT, and GPx, accompanied by a marked increase in MDA and oxidative DNA damage, reflected by elevated 8OHdG levels in both serum and BALF. Importantly, a significant compartment-specific pattern were observed between BALF and serum: SOD and GPx levels were higher in BALF, whereas 8OHdG and MDA concentrations were higher in the serum.

### Key findings and interpretation

Our study provides several novel observations that help explain the oxidative stress response following isolated severe brain injury. First, we demonstrated a compartment-specific redox pattern, with higher SOD and GPx levels in BALF and higher systemic concentrations of MDA and 8-OHdG. This pattern may indicate that pulmonary antioxidant defenses are activated early after brain injury, whereas systemic oxidative damage predominates in the circulation.

The observed association between higher PaO_2_ at the earliest time point and increased 28-day mortality warrants cautious interpretation. Higher PaO_2_ values may partly reflect more aggressive ventilatory management in patients with more severe early neurological compromise, representing a potential confounding effect rather than a causal relationship.

Thus, two competing explanations remain possible: oxygen exposure may contribute to oxidative stress, or the observed associations may reflect underlying disease severity and clinical management. Given the observational design, causality cannot be established. Future studies with appropriate adjustment for injury severity and ventilatory parameters are needed to resolve this question.

The higher antioxidant enzyme levels observed in BALF may also reflect the role of the lung as a first-line interface exposed to circulating pro-oxidant mediators released after brain injury, thereby eliciting an early compensatory response aimed at limiting local oxidative damage.

These results may reflect brain–lung cross-talk and oxygenation-associated redox alterations after severe brain injury.

In our previous study, we demonstrated significant activation of different apoptotic pathways as an important process occurring in the lungs of patients in the early phases after severe brain trauma (20).

The inverse relationship between PaO₂ and 8-OHdG concentrations suggests that lower oxygenation was associated with greater oxidative DNA damage, while higher arterial oxygen tension was associated with higher antioxidant marker concentrations.

The debate regarding oxygen supplementation in severe brain injury has been dynamic for many years, and the optimal targets for both FiO₂ and arterial oxygenation remain a subject of ongoing investigation.

The effects of different FiO₂ and PaO₂ levels require careful evaluation, given their potential influence on secondary injury mechanisms.

In this study, PaO₂ > 100 mmHg was used as a predefined exploratory threshold rather than as a conventional definition of severe hyperoxemia, intentionally chosen to explore whether elevations in arterial oxygenation within the clinically acceptable range modify the oxidative stress profile. This hypothesis-driven approach allowed for the assessment of more subtle oxygen-related redox alterations that may not be identifiable when applying the higher hyperoxia cut-offs (typically >120 mmHg) used in many previous studies.

Hyperoxemia is a common finding during the initial management of trauma patients, particularly in those who are intubated and mechanically ventilated (27).

In a large multicenter study, hyperoxia in TBI patients in the first 24 h after ICU admission was not associated with greater in-hospital mortality (28).

Controversially, Asher et al. documented that a PaO_2_ threshold between 250 and 486 mmHg during the first 72 h after brain injury was associated with improved all-cause survival in patients (29). In our cohort, PaO₂ values exceeding the predefined exploratory threshold of 100 mmHg at the earliest time point were associated with 28-day mortality in an unadjusted analysis.

Rincon et al. documented that hyperoxia in stroke-ventilated patients admitted to the ICU was independently associated with in-hospital mortality (30). Similarly, the CENTER-TBI multicenter study reported that exposure to higher PaO₂ and FiO₂ levels was associated with increased six-month mortality. Importantly, the severity of the brain injury did not change this relationship. The authors argued that the OzENTER-TBI cohort did not validate the findings because of the limited sample size. The authors concluded that worse outcomes associated with higher PaO_2_ were due to the use of higher FiO_2_ in patients with more severe injury or physiological compromise (31). The discrepancy between studies may reflect differences in patient selection, timing, and duration of oxygen exposure, or variability in defining hyperoxia thresholds. Moreover, FiO₂ titration strategies and physiological heterogeneity may have influenced the observed associations, highlighting the need for individualized oxygen therapy in neurocritical care (31). A recent updated meta-analysis by Romero-Garc et al., encompassing 66 studies and over 98,000 patients with acute brain injury, confirmed that hyperoxaemia was significantly associated with an increased risk of poor neurological outcomes (OR 1.29) and all-cause mortality (OR 1.13), particularly in subarachnoid haemorrhage and ischaemic stroke subgroups, further supporting cautious oxygenation titration in neurocritical care (32). Consistent with these findings Lalla et al. demonstrated a dose dependent association between hyperoxia and decreased favourable outcomes in mechaniclaly ventilated patients with TBI, further supporting the clinical relevance of avoiding excessive oxygen exposure in this poputation (33).

In contrast, a sub-study of the TRAUMOX2 randomised trial found no significant difference in MDA or SOD levels between a restrictive and a liberal oxygen strategy in adult trauma patients within 48 h of admission (34). This suggests that short-term oxygen exposure may not uniformly translate into measurable increases in systemic oxidative stress markers. Whether this applies to patients with pre-existing redox imbalance following brain injury, or to more prolonged exposures as in neurocritical care, remains to be established. Importantly, TRAUMOX2 enrolled a heterogeneous trauma population with an 8-h intervention window, whereas our cohort consisted exclusively of patients with isolated severe brain injury monitored over 7 days, a population in which cumulative oxidative burden and neuronal vulnerability to redox imbalance may differ substantially.

The levels of selected enzymes and their products control several metabolic processes and a variety of ROS, thereby controlling the broad aspects of a cellular system. At the mechanistic level, excessive oxygen availability can enhance reactive oxygen species (ROS) formation through mitochondrial electron leakage, NADPH oxidase activation, and Fenton-type reactions catalyzed by transition metals.

A recent review by Zeng et al. (2024), focusing on high-concentration oxygen therapy after cerebral haemorrhage, highlighted that the relationship between oxygen dose and oxidative injury is not linear: when administered within an appropriate therapeutic window, normobaric high-concentration oxygen may improve cerebral oxygenation and reduce oedema, whereas excessive or prolonged exposure risks amplifying oxidative injury through HIF-1 def activation and disruption of redox homeostasis (35). These observations are consistent with the compartment-specific and time-dependent patterns of antioxidant depletion observed in the present study.

These processes lead to the oxidative modification of lipids, proteins, and nucleic acids, ultimately impairing membrane integrity and cellular homeostasis. Mitochondrial dysfunction plays a dual role, both as a source and target of ROS, creating a self-amplifying loop of oxidative injury. Insufficient levels of antioxidant systems, such as SOD, CAT, and GPx, may further exacerbate this imbalance and propagate tissue damage.

Notably, ROS production requires O_2_, and under oxygen deprivation (hypoxia), ROS formation has been documented in several studies (in the mechanism of complex III of the mitochondrial respiratory chain or NADH accumulation) (36, 37).

A deficiency of antioxidant enzymes, such as SOD, CAT, and glutathione peroxidase (GSH-Px), and an increase in oxidative stress are observed in neurological diseases, such as Alzheimer’s disease, Parkinson’s disease, and stroke (38).

Importantly, glutathione peroxidase (GPx) operates preferentially at low H₂O₂ concentrations, owing to its high substrate affinity whereas catalase (CAT) becomes the dominant H₂O₂-scavenging enzyme when intracellular H₂O₂ levels rise (39, 40).

The divergent trajectories of these two enzymes over the first 7 days after injury may therefore reflect a time-dependent shift in the predominant H₂O₂ production pathway, rather than a uniform decline in antioxidant capacity.

The brain is vulnerable to oxidative stress damage because of the increased rate of oxidative metabolic activity and high ROS production (41). Oxidative stress is one of the major contributors to the pathophysiology of brain injury. The results of Our study are correlated with previous research that documented significant changes in antioxidants and peroxidation products in ischemic or hemorrhagic brain injury, but it showed that changes are observed in serum and may potentially act on other organs, especially the lungs (42–44). BALF analysis represents a novel aspect of this study, providing insights into the oxidative processes occurring within the lungs.

Antioxidant systems play a key role in limiting ROS-mediated toxicity and maintaining redox homeostasis. In the present study, SOD and GPx concentrations were higher in BALF than in serum, suggesting a local pulmonary antioxidant response that may be compensatory but potentially insufficient in the context of ongoing oxidative stress. Previous experimental studies have shown that hyperoxia exposure may increase the activity of antioxidant enzymes, including superoxide dismutases (45). In an animal model, the capacity of alveolar type II cells to remove extracellular H₂O₂ did not change during acute hyperoxia. However, during the second week of hyperoxia exposure, H₂O₂ production increased significantly, with alveolar macrophages rather than type II cells appearing to be the main source of H₂O₂ during prolonged exposure to high oxygen levels (46).

SOD catalyzes the dismutation of superoxide radicals into H₂O₂ and O₂ and plays an important role in antioxidant defense and redox signaling.

In the present study, SOD was measured using an ELISA kit described by the manufacturer as detecting Superoxide Dismutases (SOD), without explicit isoform-specific identification. Therefore, the measured SOD concentrations should not be interpreted as selectively reflecting SOD1. This distinction is important because SOD1 is predominantly intracellular, whereas SOD3 is the major extracellular Cu/Zn superoxide dismutase and may be particularly relevant in extracellular compartments such as serum and BALF. Accordingly, increased SOD concentrations in serum or BALF may reflect a combination of antioxidant response, extracellular SOD isoforms, and/or release of intracellular SOD from injured cells. Future studies using isoform-specific assays are needed to distinguish SOD1-related cellular injury from SOD3-mediated extracellular antioxidant defense (47). It is worth noting that lower serum SOD concentrations have previously been associated with higher inflammatory marker levels, including CRP and IL-6, and worse cognitive scores after mild acute ischemic stroke (48).

However, SOD concentrations should not be interpreted in isolation. Increased SOD activity or concentration without a proportional increase in downstream H₂O₂-detoxifying enzymes, such as CAT and GPx, may contribute to redox imbalance through H₂O₂ accumulation. This mechanism may be relevant to lung injury and acute respiratory distress syndrome (49).

The elevated SOD concentrations observed in non-survivors may therefore reflect an early but insufficient compensatory antioxidant response to overwhelming oxidative stress. This interpretation is consistent with studies in critically ill patients, in which a marked increase in SOD without a proportional rise in catalase was associated with fatal outcomes, and with data showing that higher MDA levels independently predicted 30-day mortality in severe TBI (50–52). These findings suggest that the balance between antioxidant capacity and oxidative load, rather than enzyme concentrations in isolation, may be the critical determinant of outcome.

The 8OHdG concentration likely reflects the balance between the oxidative DNA damage and repair processes. Oxidized nucleotides can be detected in various biological fluids, including bronchoalveolar BALF, and may serve as useful indicators of oxidative stress dynamics. The elevated MDA levels observed in this study further suggested lipid membrane damage resulting from excessive ROS concentrations.

In interpreting these findings, it should be noted that no clinically established reference values exist for the analyzed oxidative stress markers. Therefore, the present analysis reflects the relative differences between the groups rather than the absolute clinical assessments. The evaluated biomarkers, particularly 8OHdG, MDA, and SOD, may hold potential as adjunctive tools for assessing oxidative stress intensity in critically ill brain-injured patients. Future studies, including well-defined control populations, are warranted to establish normative ranges and facilitate clinical application. Given the dynamic nature of oxidative processes, therapeutic interventions may need to focus on maintaining redox homeostasis through personalized approaches that balance oxygen supplementation with the antioxidant capacity of individual patients, thereby reflecting both cellular and systemic metabolic demands.

## Conclusion

Severe isolated brain injury was associated with progressive systemic and pulmonary oxidative stress during the first 7 days after injury, as reflected by decreasing antioxidant marker concentrations and increasing oxidative damage marker concentrations in serum and BALF. Associations between PaO₂ and oxidative stress markers were complex and varied according to the analytical approach and biological compartment.

In an exploratory threshold-based analysis, PaO₂ values above the predefined threshold of 100 mmHg at the earliest time point were associated with higher 28-day mortality. However, this finding was not supported by a fully adjusted prognostic model including injury severity and ventilatory variables, and therefore should be interpreted cautiously as hypothesis-generating.

These results do not establish a causal relationship between supplemental oxygen, oxidative injury, or mortality. Rather, they support the need for further prospective studies incorporating predefined oxygen exposure thresholds, cumulative and time-weighted oxygenation metrics, and multivariable adjustment for injury severity and ventilatory management to clarify the role of oxygenation-associated redox alterations in severe traumatic brain injury.

### Strengths of the study

A major strength of this study is the simultaneous assessment of oxidative stress markers in serum and BALF, allowing for a direct comparison of systemic and local pulmonary redox responses in patients with isolated severe brain injury—an approach rarely reported in the literature. The longitudinal design with three standardized time points enabled evaluation of the dynamic evolution of oxidative processes. Additionally, correlating these biomarkers with PaO₂, FiO₂, imaging-based severity scales, and mortality enhances the clinical relevance and translational potential of the findings.

### Limitations

This study presents limitations such as a small group of patients and a lack of serum markers of brain injury. The reference values for 8-OHdG, MDA, SOD, CAT, and GPx1 have not yet been standardized in the clinical context. As normative clinical thresholds are not available, the findings represent relative trends rather than definitive clinical values.

It should be noted that ELISA-based measurements reflect protein concentrations rather than enzymatic activity, which may limit direct interpretation of functional antioxidant capacity. MDA is an intermediate product subject to metabolic transformation; its reduction cannot be interpreted as unequivocal stress compensation. The manufacturer’s documentation available to us did not specify whether the ELISA standards were produced in bacterial or mammalian expression systems. Therefore, no assumptions regarding the production system of the standards were made. This may limit detailed interpretation of assay specificity and comparison across different ELISA platforms. Moreover, in this study PaO₂ > 100 mmHg was used as a predefined exploratory threshold, whereas many previous clinical studies have used higher cut-off values for hyperoxemia, most commonly PaO₂ > 120 mmHg or even >200 mmHg.

This lower threshold was intentionally adopted to explore whether higher PaO₂ values within the clinically acceptable range modify the oxidative stress profile in this specific population. However, this discrepancy in the operational definition of hyperoxemia may limit the direct comparability of our results with other cohorts and could partly influence the observed associations between PaO₂ and clinical outcomes.

Additional limitations include the absence of a control group and the exploratory design without formal sample size calculation. The relatively small cohort limited the ability to perform multivariable analyses adjusting for confounders such as injury severity and ventilatory parameters. Therefore, the observed associations between PaO₂ and oxidative stress markers should be interpreted with caution and cannot establish causality. Furthermore, the assessment of oxygen exposure was based on point-in-time PaO def measurements rather than integrated time-weighted exposure, which limits the ability to assess cumulative oxidative burden. Ventilatory parameters, including PEEP, tidal volume, and PaCO def management, as well as systemic inflammatory state and haemodynamic instability, were not included as covariates in the adjusted models, which may have influenced the observed associations. Although mixed-effects models were applied to account for within-subject correlation across time points, the sample size precluded the construction of fully adjusted multivariable models incorporating injury severity scores alongside oxygenation parameters. Consequently, mortality-related findings should be regarded as exploratory and should not be interpreted as evidence that PaO₂ values above 100 mmHg independently predict mortality.

## Data Availability

The raw data supporting the conclusions of this article will be made available by the authors, without undue reservation.
