# Supplementary material for: Associations between arterial oxygenation and oxidative stress markers in serum and bronchoalveolar lavage fluid of patients with severe brain injury
Source: Front Neurol. 2026 Jul 21;17:1794885. doi: 10.3389/fneur.2026.1794885 (PMC13433365; doi:10.3389/fneur.2026.1794885)
Supplement: Supplementary file 1 [file Table_1.docx]

|  | Hydroxydeoxyguanosine (8-OHdG) | Malondialdehyde (MDA) | Superoxide Dismutases (SOD) | Catalase (CAT) | Glutathione Peroxidase 1 (GPx1) |
| --- | --- | --- | --- | --- | --- |
|  | Oxidative damage markers | | Oxidative responses markers | | |
| The data of the test and manufacturer | ELISA Kit for 8-Hydroxydeoxyguanosine (8-OHdG), Cloud-Clone Corp., USA | ELISA Kit  For Malondialdehyde (MDA), Cloud-Clone Corp., USA | ELISA Kit for Superoxide Dismutases (SOD), Cloud-Clone Corp., USA | ELISA Kit for Catalase (CAT), Cloud-Clone Corp., USA | ELISA Kit for Glutathione Peroxidase 1, Cloud-Clone Corp., USA |
| The detection range | 74.07-6.000 pg/mL | 24.69-2,000 ng/mL | 0.156-10 ng/mL | 0.312-20 ng/mL | 3.12-200 ng/mL |
| The sensitivity | 28.54 pg/mL | 8.84 ng/mL | 0.055 ng/mL | 0.119 ng/mL | 1.17 ng/mL |

TABLE 1 suplemental

The concentrations of oxidative stress markers Hydroxydeoxyguanosine (8-OHdG,) Malondialdehyde (MDA) and antioxidants: Superoxide Dismutases (SOD), Catalase (CAT), and Glutathione Peroxidase 1 (GPx1), were determined using a ready-to-use test kit according to the original manufacturer’s instructions. The data of the test manufacturer, the detection range and the sensitivity of the assays are presented in table.
