# Supplementary material for: Associations between arterial oxygenation and oxidative stress markers in serum and bronchoalveolar lavage fluid of patients with severe brain injury
Source: Front Neurol. 2026 Jul 21;17:1794885. doi: 10.3389/fneur.2026.1794885 (PMC13433365; doi:10.3389/fneur.2026.1794885)
Supplement: Supplementary file 2 [file Table_2.docx]

|  | **General** | **Survivors** | **Non-survivors** |
| --- | --- | --- | --- |
| **Number of patients** | **40** | **24** | **16** |
| **Gender** | **16 W/24M** | **7W/17M** | **9W/7M** |
| **Age** | 45 (15) | 44(17) | 47 (12) |
| **CT findings** | | | |
| **Marshal II** | 5 | 5 | 0 |
| **Marshal III** | 3 | 3 | 0 |
| **Marshal IV** | 8 | 6 | 2 |
| **Marshal V** | 19 | 10 | 9 |
| **Marshal VI** | 5 | 0 | 5 |
| **Clinical examination** | | | |
| **Glasgow Coma Score** | 4.5 (1,33) | 5.29(1,12) | 3.3 (0,47) |
| **APACHE II score** | 22 (5) | 21 (6) | 24 (5) |
| **Rotterdam CT scale** | 4(1) | 4(1) | 5(1) |
| **Lactate during admission*** | 3.2(1.37) | 2.85(1.35) | 3.73 (1.27) |
| **CRP A** | 144.15 (59.19) | 129.29 (57.88) | 166.44(55.59) |
| **CRP B** | 187.80 (46.15) | 174.29 (39.31) | 208.06(49.37) |
| **CRP C** | 196.08 (69.68) | 172.83(56.12) | 230.94(75.05) |
| **ICU length of stay*** | 32 (43) | 45 (51) | 14 (4) |
| **Ventilation days*** | 26 (34) | 36(41) | 13 (4) |
| **PaO_2_/FiO_2_ ratio at admission*** | 345.9 (116.3) | 304.7(76.3) | 407.6 (139.6) |

**Table 2 suplemental**

**Main characteristics of the patients.**

Data are presented as numbers (n) for categorical variables and as means (standard deviations) for continuous variables. The table compares demographic, radiological, and clinical parameters between survivors and non-survivors following severe isolated brain injury. *The Marshall score is presented by number of patients. ICU, intensive care unit; FiO2, fraction of inspired oxygen; PaO2, partial pressure of oxygen, * statistically significant difference between survivors and non-survivors*

Printed: Siwicka-Gieroba D, Terpilowska S, Robba C, Kotfis K, Wojcik-Zaluska A, Dabrowski W. Concentration of Apoptotic Factors in Bronchoalveolar Lavage Fluid, as Potential Brain-Lung Oxygen Relationship, Correspond to the Severity of Brain Injury. J Integr Neurosci. 2023 Mar 6;22(2):49. doi: 10.31083/j.jin2202049.
