# Supplementary material for: Associations between arterial oxygenation and oxidative stress markers in serum and bronchoalveolar lavage fluid of patients with severe brain injury
Source: Front Neurol. 2026 Jul 21;17:1794885. doi: 10.3389/fneur.2026.1794885 (PMC13433365; doi:10.3389/fneur.2026.1794885)
Supplement: Supplementary file 3 [file Table_3.docx]

**Table 3 supplemental.** Mixed-effects model results showing associations between oxidative stress markers (BALF and serum) and clinical parameters (EVLWI, FiO₂, PaO₂, PVPI), with day of measurement included as a fixed effect.

|  |  | EVLWI | | FiO2 | | PaO2 | | PVPI | |
| --- | --- | --- | --- | --- | --- | --- | --- | --- | --- |
| marker | material | estimate | p-value | estimate | p-value | estimate | p-value | estimate | p-value |
| 8OHdG | BALF | -0.0033 | 0.6837 | 0.2233 | 0.0860 | 0.0006 | 0.0629 | -0.0012 | 0.9535 |
|  | serum | 0.0160 | 0.0000 | 0.0780 | 0.2162 | -0.0002 | 0.3448 | 0.0085 | 0.4160 |
| CAT | BALF | 0.0049 | 0.4904 | 0.0951 | 0.4034 | 0.0015 | 0.0007 | 0.0015 | 0.9449 |
|  | serum | -0.0146 | 0.0624 | -0.2223 | 0.0762 | 0.0011 | 0.0210 | -0.0357 | 0.1437 |
| GPx | BALF | -0.0111 | 0.0827 | -0.0708 | 0.4891 | 0.0012 | 0.0032 | -0.0024 | 0.9035 |
|  | serum | -0.0300 | 0.0034 | -0.3141 | 0.0632 | 0.0017 | 0.0040 | -0.0148 | 0.6402 |
| MDA | BALF | 0.0373 | 0.0407 | 0.1708 | 0.5704 | 0.0012 | 0.2764 | 0.0665 | 0.2399 |
|  | serum | 0.0044 | 0.4709 | 0.1701 | 0.0772 | 0.0007 | 0.0236 | 0.0085 | 0.6198 |
| SOD | BALF | 0.0081 | 0.3696 | 0.0494 | 0.7265 | 0.0010 | 0.0653 | 0.0240 | 0.3759 |
|  | serum | -0.0010 | 0.9256 | 0.3251 | 0.0354 | 0.0016 | 0.0061 | 0.0024 | 0.9382 |
